# There and Back Again: A (Reversed) Vygotskian Perspective on Digital Socialization

**DOI:** 10.3389/fpsyg.2021.501233

**Published:** 2021-02-24

**Authors:** Maria Falikman

**Affiliations:** ^1^School of Psychology, HSE University, Moscow, Russia; ^2^Institute for Social Sciences, RANEPA, Moscow, Russia

**Keywords:** cultural-historical psychology, digital technologies, mediation, zone of proximal development, cognitive development

## Introduction

Among the most important current advances of the contemporary material and non-material culture is the development of digital technologies as new tools or instruments of the human mind. Their reciprocal influence on the human mind is, in turn, becoming one of the front edges of research in both personality and cognitive psychology and neighboring disciplines, such as philosophy of mind, neurobiology, and cultural anthropology. The unprecedented speed of technological development, together with the merging of digital and wireless technologies with human daily routines across the planet, makes this research quite complicated. One of the first-priority tasks for psychology is to develop methodology which could become a foundation for the study of co-development of human mind and digital technologies, including both common-use devices and applications for data generation, storage, and processing.

## Vygotsky's *Instrumental Method* and Mediation

Over half a century earlier, the founder of cultural-historical psychology Lev Vygotsky proposed a framework to understand cultural development of human higher mental (psychological) functions (Vygotsky, 1978; Cole, 1996). As one of the core concepts, he introduced a concept of cultural mediation as acquisition of new *cognitive tools* (such as concepts, or digits, or mnemonics) from adults within a so-called *zone of proximal development*. The latter refers to a gap between a child's unaided performance in a certain new domain and performance supported by an experienced adult. Mediation transforms the structure of human mental functions, so that psychological tools become the essential part of the entire cognitive system, and “limitlessly broadens the range of activities within which the new psychological functions may operate” (Vygotsky, 1978, p. 55).

In his paper entitled *The instrumental method in psychology*, Vygotsky wrote: “In human behavior, we can observe a number of artificial means aimed at mastering one's own psychological processes. These means can be conditionally called psychological tools or *instruments*… Psychological tools are artificial and intrinsically social, rather than natural and individual. They are aimed at controlling human behavior, no matter someone else's or one's own, just as technologies are aimed at controlling nature” (Vygotsky, 1982, vol. 1, p. 103, my translation). What is crucial, these tools emerge similarly in human evolution and in individual development and are “inherited” from social environment through the process called *socialization*. In this paper, I will demonstrate that the idea of the instrumentality of human mind might be fruitful for the understanding of our mind's transformation together with new technologies through digitally mediated socialization.

In Vygotsky's cultural-historical psychology, the acquisition of means allowing to control one's mental functions and behaviors as the core part of a child's socialization is described as *internalization*, or transformation of externalized social forms of these functions into internal. According to Vygotsky, higher psychological functions are first divided or shared between a child and an adult. What the child cannot do on her own, she can perform together with an adult who meanwhile introduces cultural tools which mediate cognition and performance (e.g., words for naming, indicating and categorizing; numbers for counting; mnemonic tools for remembering, etc.). Mental operations not yet available to a child on her own but available together with an adult define the child's zone of proximal development. Then, these culturally inherited *tools of the mind* (cf.: Bodrova and Leong, 2007) are internalized through the course of development to become tools of an individual mind.

## Vygotskian Framework In The Modern Context

In our days, the Vygotskian framework is becoming more and more popular in the developmental cognitive science. This tendency dates back to the publication of Vygotsky's *Mind and Society* in English (Vygotsky, 1978), immediately followed by a resonant article by Toulmin *Mozart in Psychology* (Toulmin et al., 1978). Vygotsky's ideas about cognitive development in general and distributed cognition in particular found further elaboration, first of all, in the area of evolutionary cognitive science, for example, in the studies of human communication, joint attention, and shared intentionality (Tomasello and Carpenter, 2007), and in the so-called *neuroarchaeology*, a new interdisciplinary research area which aims at studying how human brain and mind change through the historical development of material tools and environment (e.g., Malafouris, 2013).

Nowadays, the world and in particular technologies change faster than one could imagine, providing new forms of support for our memory, spatial navigation, attentional orienting, visual search, emphasizing an urgent need to investigate how human mind develops and changes in a culture which does not remain unchangeable (Cole and Packer, 2016), and in which digital technologies play a central role. Its current development could be best described as creating new *artifact ecologies* (Bødker and Klokmose, 2011), or “environments where multiple heterogenous technologies co-exist and are interlinked as a unified system” (Vasiliou et al., 2015, p. 55). What's remarkable about human artifact ecologies now is digitalization of social interactions, which Vygotsky considered a necessary condition of socialization. Or, to take a somewhat different perspective, from the very birth a child is embedded in a *sociotechnical system*, allowing to master “a cultural tool kit,” which nowadays includes new digital tools together with older ones, such as literacy or numeracy (Pea and Cole, 2019).

## Mediation, Internalization, and Digital Technologies

I argue that the current boost of digital technologies causes two remarkable *reversals* in the course of cognitive development as outlined in Vygotsky's “cultural-historical psychology.”

*First*, according to Vygotsky, for centuries, the trend in both individual cognitive development and the cognitive evolution as a whole was from external to internal tools of the mind, e.g., from real knots to mental notes, or from chops on the wood to mental calculations. From the cultural-historical psychology viewpoint, socialization is internalization (see also: Pea and Cole, 2019). Today, human higher psychological functions are becoming mostly externalized again due to the use of new digital tools, such as reminders, web search instead of memory search, highlighted keywords which guide our visual attention, etc. In other words, the humanity moves back from internalization to what I would call “new externalization,” with digital tools becoming an integral part of our cognitive system. In philosophy, this phenomenon has been described as Extended Cognition (Clark and Chalmers, 1998). From the psychological viewpoint, this all means reconstruction of the system of higher psychological functions through the digitally mediated activity (cf. Kaptelinin and Nardi, 2009). What is unique about digital technologies is that “extension” goes far beyond the tool (device or application) itself and is unavailable without it. In other words, such tools provide extended access rather than just support, or scaffolding. As a consequence, the borders between one's cognitive system and a technical device become blurred, with no clear understanding where, for instance, one's memory ends and a distributed world-wide web memory begins. This, in turn, influences how we remember and recall when our memory is not externally scaffolded (Ward, 2013). Such changes have also been reflected upon within the Embodied Cognition framework, as a result of “off-load of cognitive work onto the environment”(Wilson, 2002, p. 626). It is one of the reasons why this framework might be fruitfully integrated with cultural-historical approach (Zhang et al., 2018).

A similar reorganization of cognition by cultural practices has been demonstrated in multiple domains of human cognition and organization of movement, for a multitude of cultural practices, starting from counting and reading. These changes, in turn, lead to the reorganization of functional systems in the brain, which can be revealed not only by functional neuroimaging (for counting, see, e.g., Hanakawa et al., 2003), but even in the volumetric changes of specific brain structures and tracts. Surprisingly, Vygotsky discussed these neural changes driven by cultural practices over eight decades ago, when he introduced his principle of signification in human behavior: “… a man introduces artificial stimuli, signifies behavior, and by means of the signs creates new connections in the brain from outside. Admitting this, we presume a new regulatory principle of behavior, a new understanding of determination of human reactions, namely a principle of signification. A man creates externally new connections in the brain, controls his brain and thus controls his body” (Vygotsky, 1982, vol. 3, p. 91, my translation). Now, these structural changes and the human brain plasticity are becoming a major point of interest in a so-called *cultural neuroscience* (e.g., Kim and Sasaki, 2014) across a wide variety of cultural practices, such as music (Gärtner et al., 2013; etc.), chess (Hänggi et al., 2014), and many others. They have been mostly studied in adult learners, but it is more than plausible, especially in the light of association between the brain structural changes and the age of the training onset (for music, see Vaquero et al., 2016), that they start from birth, as soon as the child finds herself in the social environment forcing to acquire a variety of cultural practices.

*Second*, the zone of proximal development is also being transformed by digital devices, because children now mostly master these devices on their own, without joint activities with adults, and the adults don't even need to share their skills and experience. The situation has obviously changed during the last three decades when the zone of proximal development in computer-mediated education was extensively discussed (Crook, 1991). Moreover, sometimes children are much ahead of their parents and teachers in their use of tablets, mobile apps, etc.

To make this point more straightforward, let's consider the standard sequence of a certain cultural practice or mental tool acquisition during socialization, as outlined by Vygotsky, who has distinguished four steps in this process. First, an adult applies a practice to a child, e.g., saying “We're back home, wash your hands” or holding a child's visual attention on a way through a labyrinth in an illustrated magazine using the child's index finger. Second, a child applies it back to the adult. It's easy to imagine a child saying to her mom: “Mommy, we're back home, wash your hands!” Third, a child applies the practice to herself in a loud speech: “We're back home, I go wash my hands!” Finally, the practice becomes fully mastered by a child, or internalized, and no external mediation is needed any more: the child just goes to the bathroom to wash her hands after coming back home. What's clearly seen in this example is a common vector of the child's development: from a shared externalized function to the private internalized one. Just the same can be observed, for example, for mediated remembering or counting.

With the introduction of digital devices, the developmental trajectory of digital natives becomes less predictable for the previous generation, diverging from the above-mentioned *inter-individual to intra-individual* trend described by Vygotsky. For ages, all children were being born into a “wholly and completely socially mediated” reality, in Vygotsky's words. Now, it's also a digitally mediated reality which begins shaping a child's life quite early. According to the recent USA statistical data, by the age of 1, almost all children from low-income families were already exposed to their parents' mobile phones. By the age of 2, they used the devices without the adult involvement. By the age of 4, about 75% of children had their own mobile devices (Kabali et al., 2015).

Of course, digital devices and applications do incorporate certain social and/or cultural practices, as well as any other cultural artifact, such as a spoon, forcing a baby to use it in a certain culturally determined way, transforming her natural movements. However, with the new digital technologies, the adult is no longer necessary as an instructor, and the child's attention, memory, cognition, and activity are being shaped and organized by the interaction with the device itself.

May I emphasize that, whereas some of our cultural practices are just behavioral, the other are linked to material objects, which guide their use by new members of a certain culture. To describe this sort of guidance, Malafouris (2013) in his Material Engagement Theory of cultural evolution introduces a term *material (enactive) sign*, clearly opposing his understanding to Vygotsky's who contrasts material tools and signs: “We should not expect to find *many* similarities with tools in those means of adaptation we call signs” (Vygotsky, 1978, p. 53). For Malafouris, who criticizes the above-mentioned principle of signification and the representational nature of the first human artifacts, material sign is “a constitutive part of what it expresses” (Malafouris, 2013, p. 116), which “can be engaged in real space and time” (Malafouris, 2013, p. 117). However, this makes Malafouris' approach more complementary than opposing to Vygotsky's approach, together with their common understanding of human as an “artificial” being, ever developing through both material and social engagement (Theiner and Drain, 2017).

Extending Malafouris' ideas, I would hypothesize that the new gadgets become *material signs* of a sort for the developing generation, embodying new digital affordances, just as choppers, primitive material tools, embodied specific affordances and prompted the further actions on them at the earlier stages of human evolution. On the one hand, this matches well the proposal of radical embodied cognitive neuroscience “to think of cognitive function in the brain as context-sensitive” (Kiverstein and Miller, 2015), or driven by available affordances. On the other hand, this means that the very concept of the zone of proximal development requires reconsideration, so that it could incorporate not only human-human (child-adult), but also human-computer interaction *per se*. The more general perspective at the zone of proximal development assumes that it becomes *bidirectional*. A child might well act in the adult's “zone of proximal development,” teaching the adult to use a certain digital device or a service, with a previous step (an adult instructing a child) missing in the sequence. This transformation echoes Mead's definition of “prefigurative [cultures] in which adults learn also from their children” (Mead, 1970, p. 31).

## Conclusions

New digital technologies apparently challenge the cultural-historical approach toward cognitive development. However, the constructivist nature of this approach and the concept of cultural mediation might provide new insights on extended cognition and its evolution.

## Author Contributions

The author confirms being the sole contributor of this work and has approved it for publication.

## Conflict of Interest

The author declares that the research was conducted in the absence of any commercial or financial relationships that could be construed as a potential conflict of interest.
